# Parasitoid wasp venom manipulates host innate behavior via subtype-specific dopamine receptor activation

**DOI:** 10.1242/jeb.243674

**Published:** 2022-03-23

**Authors:** Stefania Nordio, Maayan Kaiser, Michael E. Adams, Frederic Libersat

**Affiliations:** 1 Department of Life Sciences, Ben-Gurion University of the Negev, P.O. Box 653, Beer Sheva 84105, Israel; 2 Zlotowski Center for Neuroscience, Ben-Gurion University of the Negev, P.O. Box 653, Beer Sheva 84105, Israel; 3 Department of Entomology, University of California, Riverside, CA 92521, USA; 4 Department of Molecular, Cell, and Systems Biology, University of California, Riverside, CA 92521, USA

**Keywords:** Venom, Dopamine, Central complex, D1-like receptor, D2-like receptor, Grooming

## Abstract

The subjugation strategy employed by the jewel wasp is unique in that it manipulates the behavior of its host, the American cockroach, rather than inducing outright paralysis. Upon envenomation directly into the central complex (CX), a command center in the brain for motor behavior, the stung cockroach initially engages in intense grooming behavior, then falls into a lethargic sleep-like state referred to as hypokinesia. Behavioral changes evoked by the sting are due at least in part to the presence of the neurotransmitter dopamine in the venom. In insects, dopamine receptors are classified as two families, the D1-like and the D2-like receptors. However, specific roles played by dopamine receptor subtypes in venom-induced behavioral manipulation by the jewel wasp remain largely unknown. In the present study, we used a pharmacological approach to investigate roles of D1-like and D2-like receptors in behaviors exhibited by stung cockroaches, focusing on grooming. Specifically, we assessed behavioral outcomes of focal CX injections of dopamine receptor agonists and antagonists. Both specific and non-specific compounds were used. Our results strongly implicate D1-like dopamine receptors in venom-induced grooming. Regarding induction of hypokinesia, our findings demonstrate that dopamine signaling is necessary for induction of long-lasting hypokinesia caused by brain envenomation.

## Abstract (precis)

**Highlighted Article:** Subtype-specific dopamine receptors are involved in the manipulation of host behavior by the parasitoid jewel wasp.

## INTRODUCTION

The parasitoid jewel wasp (*Ampulex compressa*) manipulates its host the American cockroach (*Periplaneta americana*) through a unique envenomation strategy aimed at hijacking its behavior. This is accomplished through injection of venom directly into cockroach head ganglia (Libersat and Gal, 2014). Typically, parasitoid wasps use their venom for host paralysis to provide a food source for their developing larva (Hughes and Libersat, 2018); instead, *Ampulex compressa* manipulates the central neuronal circuitry of host head ganglia. The wasp injects venom into both head ganglia, namely the brain (or cerebral ganglia) and gnathal ganglion (GNG). In particular, after seizing the cockroach pronotum with its mandibles, the wasp injects its venom into the central complex (CX), a ‘higher center’ known to regulate motor behaviors (Guo and Ritzmann, 2013; Kaiser and Libersat, 2015; Martin et al., 2015). The CX alone is necessary for the initiation of spontaneous walking, since focal injection of procaine into the CX is sufficient to induce the decrease in spontaneous walking (Kaiser and Libersat, 2015). The stung animal manifests intense grooming behavior for roughly 25 min before entering a ‘lethargic’ hypokinetic state characterized by passivity and compliance to handling by the wasp. Interestingly, injection of reserpine or dopamine into the hemocoel induces prolonged grooming in cockroaches (Weisel-Eichler et al., 1999). Whether venom injection into the CX is necessary for induction of grooming has not been tested and will be addressed in the present study. Finally, yet importantly, biochemical analysis of the venom shows the presence of dopamine (Weisel-Eichler and Libersat, 2002; Banks and Adams, 2012). Hence, it is reasonable to associate dopamine (DA) with venom-evoked behavioral manipulation and consequently activation of dopamine receptors (DARs). DA acts on two rhodopsin-like G protein-coupled DAR subfamilies. In mammals, they are classified as D1-like (D1 and D5) and D2-like (D2, D3 and D4) receptors. In insects, D1-like (DOP1) and D2-like (DOP3) receptors have been characterized in the fruit fly (*Drosophila melanogaster*) and honeybee (*Apis mellifera*), among others (Verlinden, 2018). D1-like receptors activate adenylyl cyclase, whereas members of the D2 subfamily either inhibit adenylyl cyclase or interact with a different intracellular second messenger system. This results in increased cAMP levels following D1-like receptor activation and a decrease of this second messenger after D2-like receptor activation (Beggs et al., 2005; Watanabe et al., 2013). A number of studies in both insects and rodents suggest that grooming is driven by D1-mediated signaling, whereas D2 receptors modulate locomotion (Starr and Starr, 1986; Andretic et al., 2005; Draper et al., 2007; O'Sullivan et al., 2010; Pitmon et al., 2016). However, specific functional roles for D1-like or D2-like receptors have not yet been demonstrated in venom-induced behavioral manipulation of cockroaches by the jewel wasp. In the present study, we used a pharmacological approach to unravel involvement of D1-like versus D2-like receptors in venom-induced behavioral manipulation of the cockroach, focusing on grooming. In particular, we employed focal injection of specific and non-specific agonists and antagonists of D1-like and D2-like receptors directly into the CX of cockroaches followed by detailed behavioral analysis.

## MATERIALS AND METHODS

### Animals

Cockroaches [*Periplaneta americana* (Linnaeus 1758)] were raised in plastic containers (50×50×70 cm) at 27°C under a 12 h:12 h light:dark cycle with water and food (cat chow) *ad libitum*. Wasps [*Ampulex compressa* (Fabricius 1781)] were raised in Perspex cages (40×50×50 cm) at ambient temperature of 30°C and humidity of 40% under a 12 h:12 h light:dark cycle. They were provided with water and honey *ad libitum*. All experiments performed comply with Principles of Animal Care (NIH publication no. 86-23, revised in 1985) and with the current laws of the State of Israel.

### Behavior

Spontaneous grooming and locomotory behaviors of adult male cockroaches were measured in a circular arena with a radius of 30 cm. Two stopwatches were used to measure duration of spontaneous walking and spontaneous grooming in bouts of 10 min for a total of 30 min. In every experiment, spontaneous behavior was always observed before (baseline) and after treatment. Escape behavior was measured in a circular arena with a total radius of 30 cm divided into sectors with four concentric circles having radii of 5, 15, 25 and 30 cm. Cockroaches were placed in the center of the arena and stimulated with a soft paintbrush. The escape response was calculated by measuring distance traveled in centimeters from the center to the end of a continuous running bout. Escape distance was then binned into circles with radii of 5, 15, 25 and 30 cm. Escape behavior was tested in three trials spaced by 10 min.

### Injection

CX injections were performed as described in Kaiser and Libersat (2015). Cockroaches were anesthetized with carbon dioxide (CO_2_) for a few seconds, then placed on a Petri dish covered in clay. The body and antenna were fastened with clay and the neck was gently tied with a thread to reduce hemolymph flow to the head capsule. A small flap in cuticle between the ocelli was cut to expose the brain. A glass capillary injection needle connected to a Nanoject™ (Drummond Scientific) nanoliter injector was then inserted in the center of the brain targeting the CX and 2×9 nl of drug were injected. The small flap of the cuticle was then closed and sealed off with wax.

### Histology

After behavioral testing, accuracy of injection was verified histologically. First, the cockroach head was removed and fixed overnight in 10% formalin (Sigma, Israel). Then the brain was removed from the head, embedded in 6% agar in saline consisting of the following components (in mmol l^−1^): NaCl 214, KCl 3.1, CaCl_2_ 9, sucrose 50, HEPES buffer 5 (Sigma, Israel), and sliced into 60 µm sections with a vibratome (Leica VT 1000S). Janus Green tracer (0.5% Janus Green in saline, Sigma) was co-injected as a marker to verify accuracy of injection.

### Surgery

To investigate whether the CX of the brain or the GNG of cockroaches triggers venom-evoked responses, the circumesophageal connectives (CirCs) between these two ganglia were crushed. Crushing the CirC removes descending activity from the brain (and the CX) and therefore can be used to test the role of the GNG alone in the venom induced grooming behavior. Procedurally, cockroaches were anesthetized with CO_2_ for a few seconds, then placed on a Petri dish covered in clay. The body and antenna were fastened with clay and the neck was tied with a thread to reduce hemolymph flow into the head capsule. A small flap of cuticle under the ocelli was cut to expose part of the brain. Using forceps, connections between brain and GNG were crushed. The cuticle was closed subsequently and sealed with wax. Cockroaches were then allowed to recover for 1 h.

### Pharmacology

All drugs were injected directly into the CX following the procedure described above under ‘Injection’ above.

#### EEDQ

2-Ethoxy-1-ethoxycarbonyl-1, 2-dihydroquinoline (Sigma), an irreversible broad-spectrum antagonist of D1 and D2 receptors (Neisewander et al., 1995), was injected directly into the CX (18 nl EEDQ 0.01 mmol l^−1^). In the first experiment, cockroaches were divided into three groups of 10 individuals each. In each group, spontaneous behavior was evaluated at different time intervals (6, 12 and 24 h) after EEDQ injection. In the second experiment, cockroaches injected with EEDQ were divided into three groups, then subjected to a wasp sting 6, 12 and 24 h after injection. The behavioral study was performed immediately after the sting. In the last experiment, cockroach escape behavior was tested 1 h after EEDQ injection. This behavior was then compared with the escape behavior of stung and EEDQ-injected cockroaches.

#### Flupentixol

Cis-(Z)-flupenthixol dihydrochloride (Sigma), a reversible antagonist of D1 and D2 dopamine receptors (Notman and Downer, 1987) was injected (18 nl, 0.01 mol l^−1^) into the CX immediately prior to EEDQ injection (18 nl EEDQ 0.01 mmol l^−1^). Cockroach behavior was evaluated 6 h after injection (*n*=12). Cockroaches were then subjected to the wasp sting and spontaneous behavior (walking and grooming) was quantified again.

#### SKF38393

(±)-SKF-38393 hydrochloride (Sigma), a specific D1 receptor agonist (Guo et al., 2015), was injected (18 nl, 10^−7^ mol l^−1^) into the CX (*n*=12). Spontaneous behavior was evaluated 2 h after injection.

#### Bromocriptine

2-Bromo-α-ergocryptine methanesulfonate salt (Sigma), a specific D2 receptor agonist (Vickrey and Venton, 2011; Guo et al., 2015), was injected (18 nl, 10^−7^ mol l^−1^) into the CX of cockroaches (*n*=12). Spontaneous behavior was evaluated 2 h after injection.

#### SCH23390

R (+)-SCH-23390 hydrochloride (Sigma), a specific reversible D1 dopamine receptor antagonist (Guo et al., 2015) was injected (18 nl, 5 mmol l^−1^) before EEDQ (18 nl EEDQ 0,01 mmol l^−1^). In this experiment, spontaneous behavior was evaluated 6 h after injection. Thereafter, cockroaches were subjected to a wasp sting and spontaneous behavior was quantified again.

#### S-sulpiride

(S)-(−)-Sulpiride (Sigma), a specific reversible D2 dopamine receptor antagonist (Guo et al., 2015) was injected (18 nl, 5 mmol l^−1^) before EEDQ (18 nl EEDQ 0.01 mmol l^−1^). In this experiment, spontaneous behavior was studied 6 h after injection. Thereafter, cockroaches were subjected to a wasp sting and spontaneous behavior was quantified again.

### Statistical analysis

All statistical analyses were performed in RStudio (https://www.rstudio.com/). Results are expressed as means±s.e.m. Mean differences between two groups were examined by parametric *t*-test and paired *t*-test and by non-parametric Mann–Whitney and Wilcoxon tests.

## RESULTS

### Which head ganglion is targeted for venom-evoked behavioral modification?

**Fig. 1.**
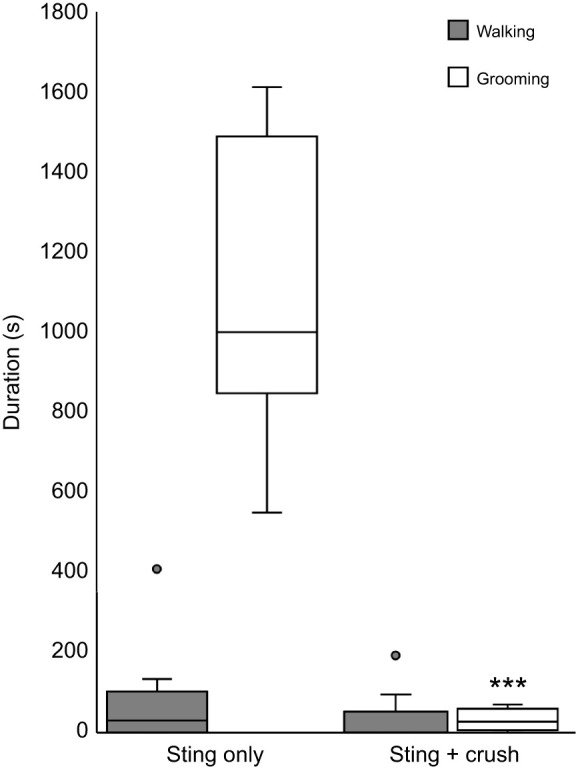
**Grooming behavior is significantly decreased in cockroaches stung by parasitic wasps when circumesophageal connectives are crushed.** Spontaneous behavior of cockroaches with crushed connectives between the brain and the GNG is compared with behavior in intact cockroaches after both groups were stung. The boxplots represent the median (white or black line), the interquartile range of duration (seconds) of walking and grooming and the eventual outliers (gray dots). In each group *n*=12 cockroaches; ***P<0.001, *t*-test.

Since the wasp injects venom into both head ganglia (the brain and GNG), we first investigated whether the CX of the brain or the GNG of cockroaches triggers the venom-evoked response. As a first approach to this question, circumesophageal connectives (CirC) between the brain and GNG (Fig. 1) were crushed. After surgery, cockroaches were subjected to a wasp sting and spontaneous behavior was studied. Insects in general and cockroaches in particular exhibit long bouts of walking activity following crush of the circumesophageal connectives (Kien, 1983; Bässler, 1983; Gal and Libersat, 2006). Unlike control stung cockroaches with intact connections between head ganglia, venom-evoked grooming was significantly reduced following crush of the CirC (paired *t*-test, *P*<0.001). From this experiment, we conclude that the CX has a major role in the venom-evoked grooming response. Subsequent pharmacological experiments described below targeted this brain region to elucidate involvement of dopamine receptors in the grooming response. In addition, we evaluated the role of dopamine receptors in long-lasting hypokinesia evoked by the wasp sting.

### Spontaneous grooming augmented by D1 receptor agonism

**Fig. 2.**
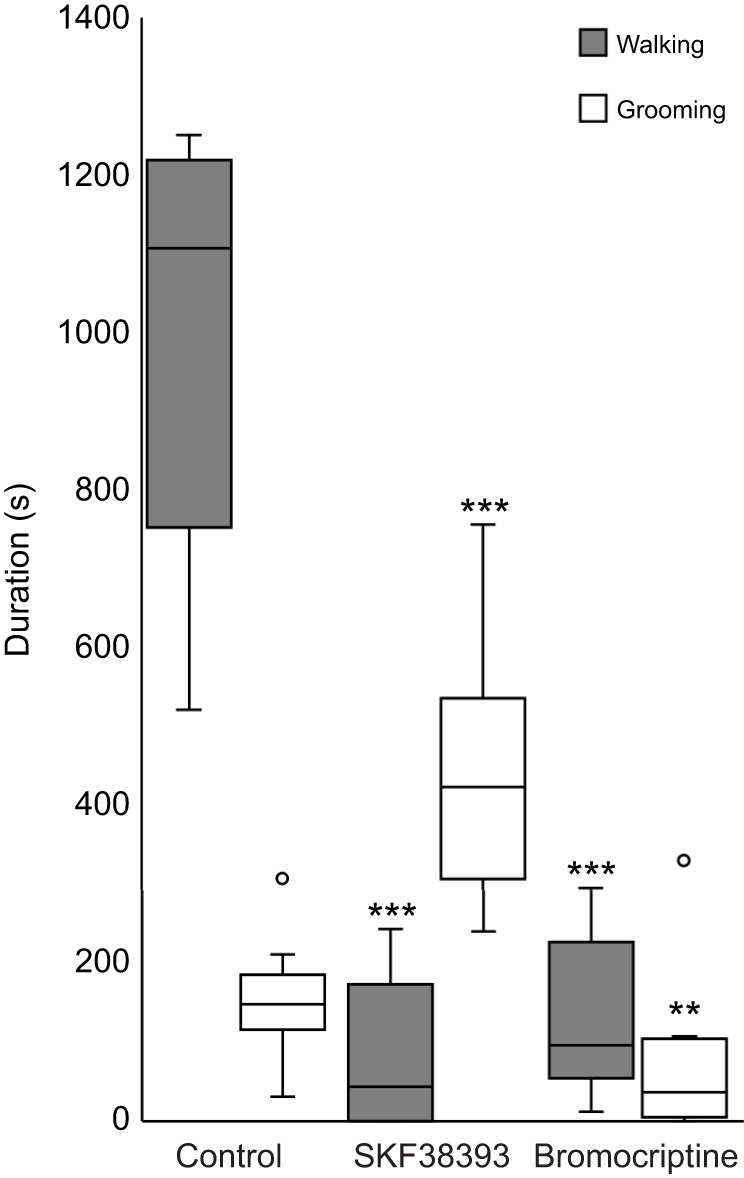
**D1 agonist injection increases spontaneous grooming duration in unstung cockroaches.** Walking and grooming behavior in cockroaches injected with D1 (SKF38393) or D2 (Bromocriptine) specific agonist is compared with that in uninjected control cockroaches. The boxplots represent the median (white or black line), the interquartile range of duration (seconds) of walking and grooming and the eventual outliers (open dots). In each group *n*=12 cockroaches; ***P<0.001, **P<0.01, paired *t*-test.

We tested whether dopamine signaling is involved in spontaneous grooming and walking by injecting selective DAR agonists into the CX (Fig. 2). Actions of the D1-like receptor agonist SKF38393 were tested first (*n*=12). Thirty minutes after injection, the duration of spontaneous grooming duration increased significantly compared with grooming duration before injection (paired *t*-test, *P*<0.001). By contrast, walking duration decreased (paired *t*-test, *P*<0.001) compared with walking duration before injection.

In the second experiment, CX injection of bromocriptine, a specific agonist of D2-like receptors, was performed on 12 cockroaches. Thirty minutes after injection, the duration of spontaneous grooming decreased slightly (paired *t*-test, *P*<0.01), whereas walking duration decreased drastically (paired *t*-test, *P*<0.001). These experiments indicate that D1-like, but not D2-like, receptors drive grooming behavior. In contrast, activation of both receptor subtypes suppresses spontaneous locomotory behavior.

### Spontaneous grooming suppressed by DAR antagonism

**Fig. 3.**
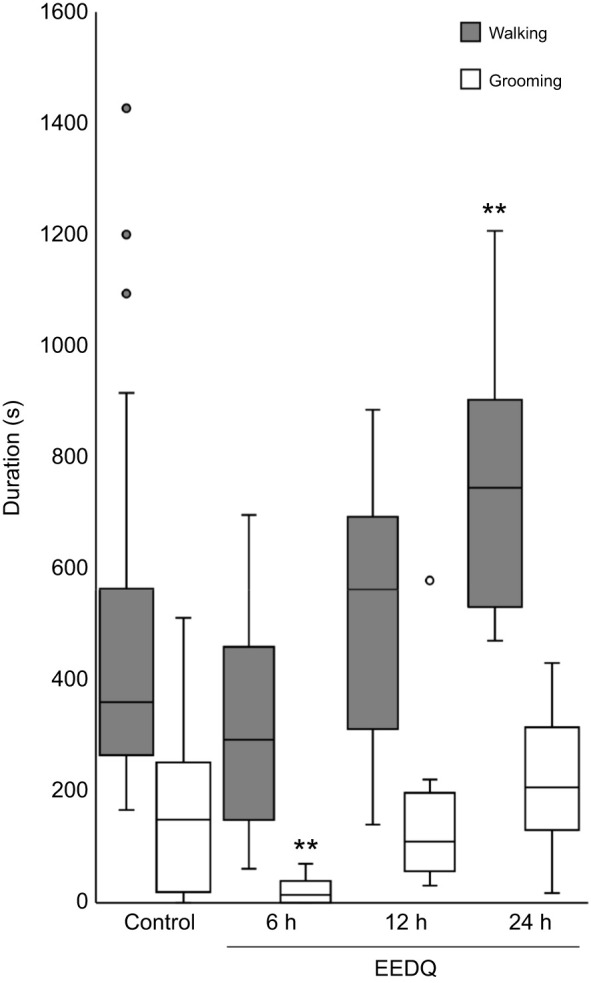
**EEDQ suppresses spontaneous grooming behavior in unstung cockroaches.** Spontaneous walking and grooming behavior measured in uninjected control animals and at three different time points after EEDQ injection: 6, 12 and 24. The boxplots represent the median (white or black line), the interquartile range of duration (seconds) of walking and grooming and the eventual outliers (gray full dots and open dots). In each group *n*=10 cockroaches; **P<0.01, paired *t*-test.

EEDQ is a broad spectrum irreversible antagonist of both D1-like and D2-like receptors in insects (Granger et al., 2000) and vertebrate D1 and D2 receptors (Hamblin and Creese, 1983). We investigated whether EEDQ injection into the CX affects spontaneous grooming. Cockroaches were divided into three groups, whereby spontaneous behavior of each group (*n*=10) was measured at three different time points after EEDQ injection: 6, 12 and 24 h, respectively (Fig. 3). When examined 6 h after EEDQ injection, cockroaches showed a significant decrease in spontaneous grooming duration (paired *t*-test, *P*=0.017), but spontaneous walking was not affected significantly. Cockroaches observed 12 h after injection presented no significant differences in either spontaneous walking or grooming compared with behavior pre-injection. Observations made 24 h after EEDQ injection revealed a significant increase in spontaneous walking duration (paired *t*-test, *P*=0.009), but no difference in grooming duration. From these experiments, we conclude that EEDQ antagonism of DAR suppresses spontaneous grooming for at least 6 h and increases spontaneous walking, but only after a delay of between 12 and 24 h.

### Venom-evoked grooming suppressed by DAR antagonism

**Fig. 4.**
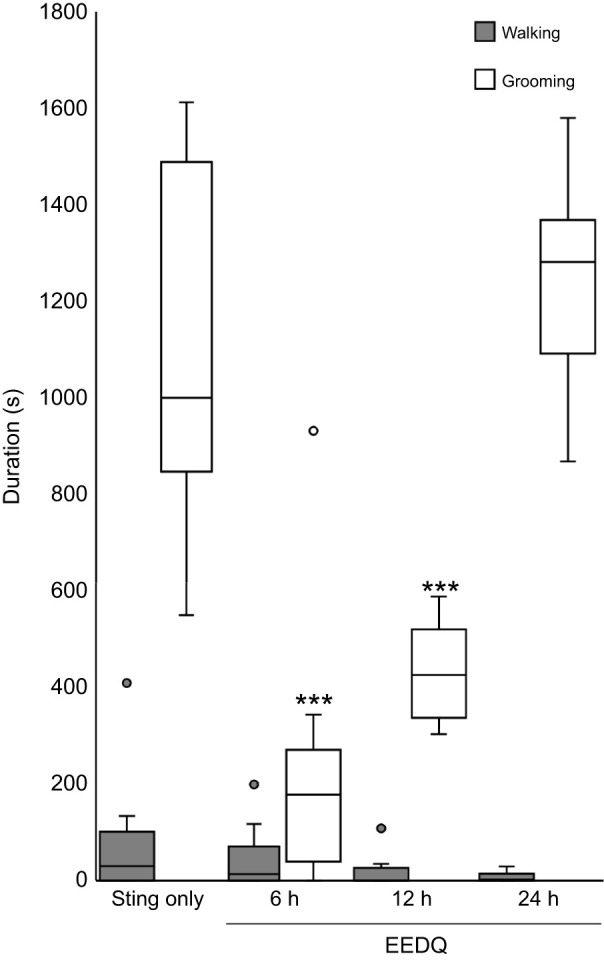
**EEDQ suppresses venom-evoked grooming behavior.** Walking and grooming behavior in control ‘sting only’ cockroaches and three different groups of EEDQ-injected animals stung at 6, 12 and 24 h after injection. The boxplots represent the median (white or black line), the interquartile range of duration (seconds) of walking and grooming and the eventual outliers (gray full dots and open dots). In each group, *n*=10 cockroaches; ***P<0.001, paired *t*-test.

To verify dependence of venom-evoked behaviors on dopamine signaling, cockroaches (*n*=30) were subjected to CX injections of EEDQ prior to a sting. Cockroaches were divided into three groups (*n*=10) according to timing of the sting following EEDQ injection: 6, 12 and 24 h, respectively (Fig. 4). Cockroaches stung 6 h after EEDQ injection showed a highly significant decrease in grooming duration (paired *t*-test, *P*<0.001) compared with non-EEDQ injected stung cockroaches. In contrast, no significant difference in walking duration was observed at this time point. Animals stung 12 h after EEDQ injection also exhibited a highly significant reduction in grooming (paired *t*-test, *P*<0.001). Again, spontaneous walking duration in this treated group did not differ from that of non-injected stung cockroaches. Twenty-four hours after EEDQ injection, grooming duration of stung cockroaches did not differ from non-injected stung cockroaches. Likewise, walking duration in these EEDQ-treated animals was not different to that of non-injected stung cockroaches. From these experiments, we conclude that DARs mediate venom-evoked behavior and, in particular, EEDQ antagonism of DARs suppresses grooming behavior typically observed in stung cockroaches 6 h after EEDQ treatment, an effect that dissipates within 12 h.

### Flupenthixol protects venom-induced grooming in EEDQ-treated animals

**Fig. 5.**
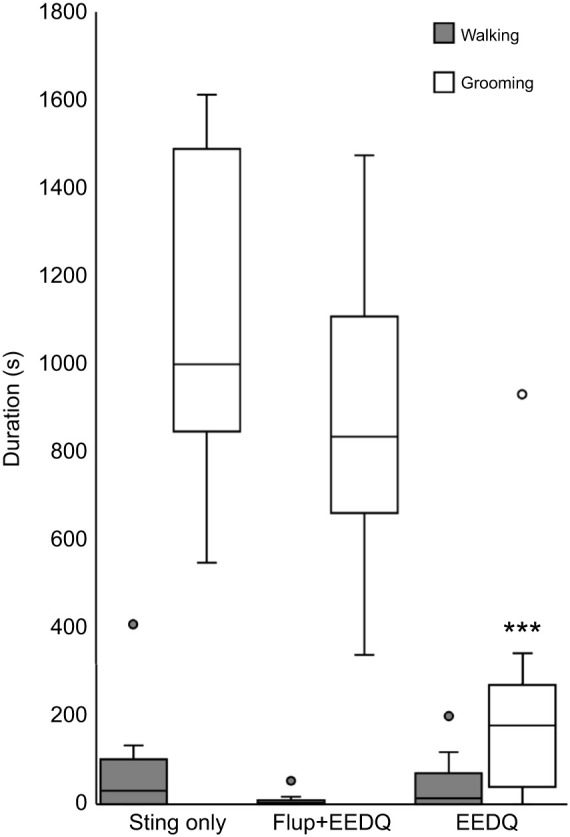
**Flupenthixol protects venom-induced grooming in EEDQ-treated cockroaches.** Walking and grooming behavior in control ‘sting only’ cockroaches compared with cockroaches stung and injected with flupentixol+EEDQ or EEDQ alone. The boxplots represent the median (black line), the interquartile range of duration (seconds) of walking and grooming and the eventual outliers (gray and white dots). Control and Flup+EEDQ, *n*=12; EEDQ only, *n*=10 cockroaches; ***P<0.001, *t*-test.

We then asked whether protection of DARs from irreversible block by EEDQ could rescue normally observed venom-evoked grooming. Such protection was provided by pre-treatment of stung, EEDQ-injected cockroaches with the broad spectrum, but reversible DAR antagonist flupenthixol. Indeed, stung cockroaches injected with flupenthixol briefly prior to EEDQ injection showed normal post-sting grooming when stung 6 h after treatment (Fig. 5). In contrast, stung cockroaches injected with EEDQ alone showed significant reduction in grooming duration (*t*-test, *P*<0.001). From these experiments, we conclude that protection of DARs from irreversible EEDQ block restores venom-evoked grooming, providing further support for the critical involvement of DARs in venom-evoked behavior. However, walking duration is not affected by these treatments.

### Venom-induced grooming depends on D1-like receptor activation

**Fig. 6.**
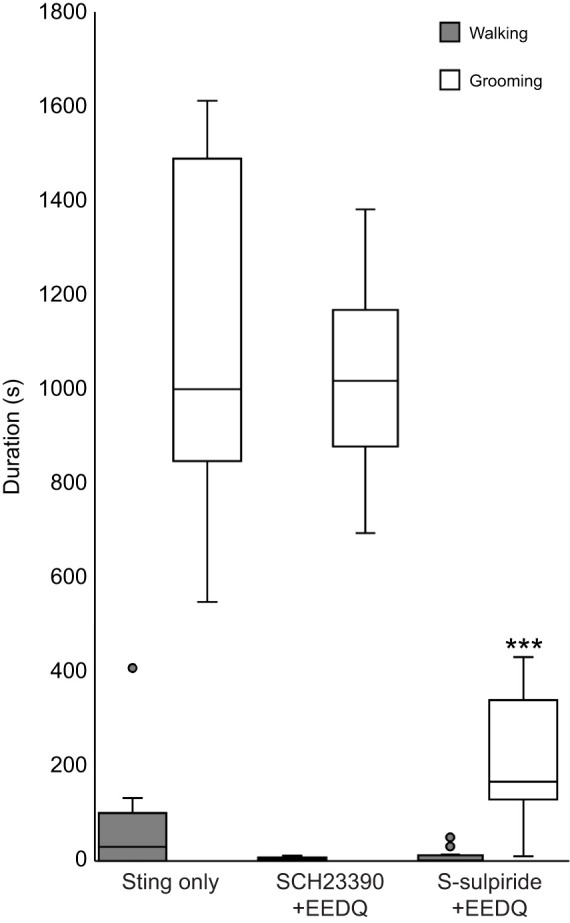
**D1 but not D2 antagonist protects venom-induced grooming in EEDQ-treated cockroaches.** Spontaneous behavior of cockroaches injected and then stung compared with control ‘sting only’ animals. D1 (SCH23390) or D2 (Sulpiride) specific antagonists were injected immediately before EEDQ injection. The boxplots represent the median (black line), the interquartile range of duration (seconds) of walking and grooming and the eventual outliers (gray full dots). In each group, *n*=12 cockroaches; ***P<0.001, *t*-test.

To further investigate roles of D1-like and D2-like receptors in venom-induced behavioral changes, DAR-specific antagonists were injected into the CX of cockroaches prior to the sting (Fig. 6). Injection of the reversible D1 antagonist SCH23390 prior to EEDQ treatment should protect D1-like receptors, resulting in selective block of D2-like receptors. Six hours later, cockroaches were subjected to a wasp sting and subsequent behaviors were quantified. Both walking and grooming behavior showed no significant difference from behaviors of non-injected stung cockroaches (Fig. 6). To protect D2-like receptors from EEDQ disruption, the specific and reversible D2-like receptor antagonist S-sulpiride was injected prior to EEDQ treatment. Six hours later, cockroaches were subjected to a wasp sting followed by behavioral analysis. We found that grooming duration was significantly reduced compared with the venom-evoked grooming duration (*t*-test, *P*<0.001), whereas walking duration was unaffected compared with non-injected stung cockroaches. These experiments provide further evidence that venom-induced grooming behavior is dependent on D1-like receptor signaling.

### EEDQ injection shortens the long-term effect of the venom on escape behavior

**Fig. 7.**
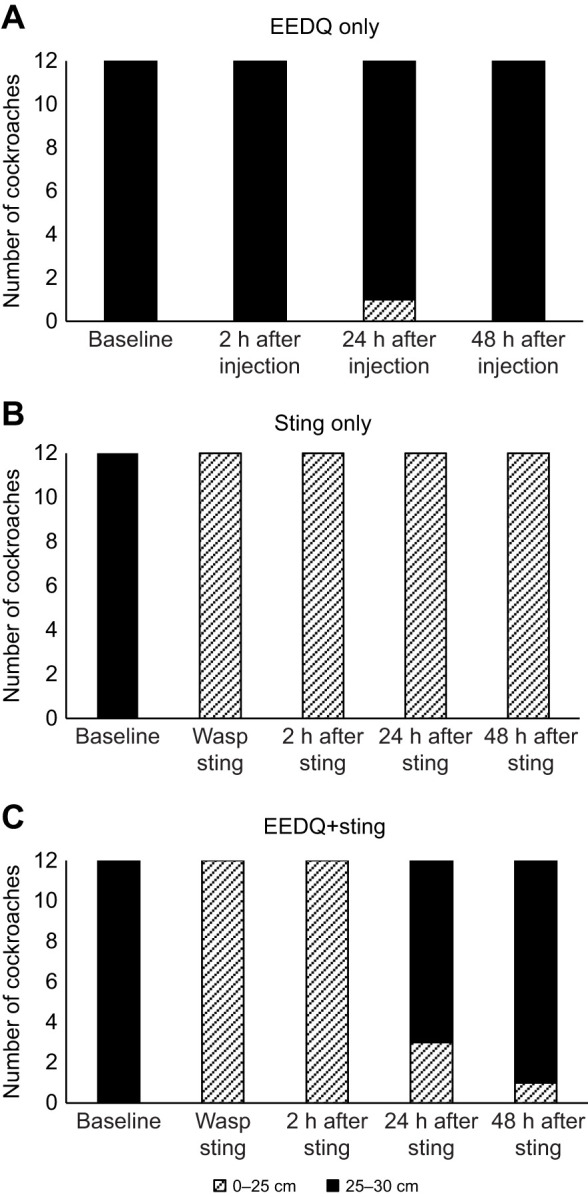
**EEDQ injection reduces the long-term effect of the venom on escape behavior.** Escape behavior in three groups of cockroaches: (A) non-stung cockroaches injected with EEDQ, (B) stung cockroaches and (C) EEDQ-injected and stung cockroaches. The escape response was measured at different time-points in an arena binned in two circles according to the size of the radius (0–25 and 25–30 cm). In each group, *n*=12; ***P<0.001, *P<0.05, paired *t*-test.

So far, evidence presented in this study strongly implicates D1-like DARs in venom-induced grooming. Since grooming is followed by a long-lasting hypokinetic state, we investigated the possible involvement of DARs in this next behavioral manipulation. This long-lasting hypokinetic state was shown to be reversible and lasts 5–7 days. To test whether DARs are involved in the long-term effect of venom on escape behavior, we used EEDQ again to remove DARs prior to a wasp sting. Then, we quantified escape responses of three groups of cockroaches at different time points up to 48 h after the wasp sting. The escape distance of cockroaches was binned into two distance ranges: 0 to 15 cm, which corresponds to a startle response, and 25 to 30 cm (the edge of the arena), which corresponds to a full escape running response (Fig. 7). The startle and escape behaviors of 3 groups of cockroaches were measured: (1) injected with EEDQ, (2) EEDQ-injected and then stung briefly after recovery from the procedure (roughly 30 min), and (3) stung only. At all the time points after baseline, stung cockroaches showed no full escape response after the wasp sting as expected (paired *t*-test, *P*<0.001) (Fig. 7). EEDQ alone had no effect on the escape behavior of cockroaches. Injection of EEDQ prior to wasp sting had no significant effect on escape behavior 2 h after the sting. In other words, these cockroaches behaved as if they had been just stung by a wasp. However, when testing these cockroaches 24 and 48 h after EEDQ injection and sting, they showed normal escape behavior. Hence, removal of DARs through EEDQ treatment shortened the long-lasting effect of the sting from roughly 5–7 days to 1–2 days. From this experiment, we conclude that DAR-mediated signaling is necessary for venom-induced long-term hypokinesia.

## DISCUSSION

One hallmark of venom-induced behavioral manipulation of cockroaches stung by the jewel wasp is intense grooming. Such uninterrupted grooming for roughly 25 min is due to presence of the neurotransmitter DA in the venom (Weisel-Eichler et al., 1999; Banks and Adams, 2012). This monoamine plays a broad range of functional roles in the control of movement, reward, motivation, arousal and memory in both insects and mammals (Verlinden, 2018). In addition, it is essential for melanisation and sclerotisation of the exoskeleton in insects. Functions of dopamine relevant to the present study are its regulation of locomotion and grooming (Banks and Adams, 2012; Daiane Stürmer et al., 2014). The action of dopamine is mediated by the rhodopsin-like family of GPCRs (Bockaert and Pin, 1999). In mammals, dopamine receptors are classified into two groups: the D1 and the D2 receptors. D1 receptors (including D1 and D5) activate the adenylyl cyclase leading to increased cAMP levels in cellular targets. D2 receptors represented by three subtypes (D2, D3 and D4), inhibit adenylyl cyclase, leading to decreased levels of cAMP (Missale et al., 1998).

In invertebrates, the D1-like (DOP1) and D2-like (DOP3) receptors have also been characterized with similar and conserved features in *Drosophila*, *A. mellifera*, *Caenorhabditis elegans* and others (Beggs et al., 2005; Karam et al., 2020). DOP1 receptors increase intracellular cAMP levels upon DA binding via a signaling cascade similar to the vertebrate D1-like receptors. The DOP3 group is more related to the vertebrate D2-like receptors, reducing cAMP levels upon agonist binding (Mustard et al., 2005; Verlinden, 2018). With this in mind, the goal of the present study was to unravel the specific roles of dopamine receptors in cockroach cerebral ganglia in host behavioral manipulation by the jewel wasp, focusing on venom-induced grooming.

The actions of dopamine are mediated via G protein-coupled receptors (GPCRs). Dopamine receptors are members of the rhodopsin-like family of GPCRs, which are characterized by seven trans-membrane spanning domains that contain the ligand-binding site, an extracellular amino-terminus, and an intracellular carboxyl-tail (Bockaert and Pin, 1999). The jewel wasp immobilizes its host, the American cockroach, by stinging directly into its head capsule, targeting both the brain and GNG. These ganglia, which are connected through the CirC, modulate locomotion and grooming (Gal and Libersat, 2006; Emanuel et al., 2020). More specifically, envenomation of the brain is focused on a precise area: the CX, which comprises four distinct, layered neuropiles and is involved in sensory processing and modulation of motor behaviors (Pfeiffer and Homberg, 2014; Guo and Ritzmann, 2013). Sensory information processed in the CX is then transferred to monoaminergic descending neurons (Okada et al., 2003; Hsu and Bhandawat, 2016). After the wasp sting, cockroaches perform intense venom-evoked grooming followed by a long-lasting hypokinetic state characterized by a significant decrease in walking and escape behavior. Venom-induced grooming seems to be primarily mediated by the CX. Unlike the control group, stung cockroaches with disrupted connections between the brain and the GNG exhibit a significant decrease in venom-evoked grooming. The effect is not due to a general decrease in cockroach stamina, but rather on the motivation of the wasp to sting. In fact, it was shown that insects with severed CirC exhibit a drastic increase in spontaneous walking (Bässler, 1983; Kien, 1983; Ridgel and Ritzmann, 2005; Gal and Libersat, 2006). Hence, our findings can be interpreted as follows: injection of venom into the CX of the brain is critical to induce grooming.

Characterization of DAR involvement in post-envenomation grooming relied heavily on use of EEDQ. This agent is a potent dopaminergic neurotoxin that binds irreversibly to vertebrate D1 and D2 receptors, inducing a marked decrease in DAR density in the striatum (Hamblin and Creese, 1983). Results of the present study indicate that EEDQ reduces spontaneous grooming behavior. Moreover, the effect of EEDQ lasts for at least 6 h, after which time normal spontaneous behavior is restored. This is in agreement with the effect of EEDQ injection reported in rats, whereby DAR density in the striatum decreases over a period of 24 h, after which it is restored to normal levels (Hamblin and Creese, 1983). Furthermore, infusion of EEDQ into the lateral caudate putamen attenuates D1 agonist-induced grooming (Neisewander et al., 1995). In the present study, we show that EEDQ likewise suppresses venom-evoked grooming behavior. Moreover, typical venom-evoked grooming behavior is restored once DARs are protected by the reversible broad spectrum DAR antagonist flupenthixol injected prior to EEDQ (Notman and Downer, 1987; Hess et al., 1988).

Our pharmacological approach using specific DAR agonists and antagonists in cockroaches reveals the contribution of D1-like receptors in venom-induced grooming. Increased grooming behavior evoked by the specific D1-like agonist SKF38393 was not observed in cockroaches subjected to the D2-like agonist bromocriptine. The same hypothesis was supported by experiments performed in rats and insects, where SKF38393 was shown to stimulate adenylyl cyclase and produce enhanced grooming (Clark and White, 1987; Stoessl, 1994; Pitmon et al., 2016). Bromocriptine, in contrast, seems to decrease levels of intracellular cAMP by virtue of its potent and effective properties as an agonist of insect DOP3 (D2-like) receptors (Verlinden et al., 2015).

We interpret actions of these agents on walking behavior as follows. After D1 agonist injections, walking behavior significantly decreased. This is not surprising, since grooming increased and both behaviors are mutually exclusive. After D2 agonist injections, walking behavior also significantly decreased with no accompanying increase in grooming. One possible explanation is that overloading the DARs with DA agonist leads to desensitization of post-synaptic DARs. Another non-exclusive alternative is that the DA agonist binds to autoreceptors of the presynaptic membrane causing a decrease in DA release and reduction of walking behavior (Qi and Lee, 2014). Vickrey and Venton (2011) reported that the D2 agonist bromocriptine decreased stimulated dopamine release in the *Drosophila* brain by activating the D2-like receptor DD2R. (Vickrey and Venton, 2011). Yet, the most likely explanation is that the venom has long-term effects on postsynaptic neuronal excitability aside from activation of DARs.

To explore further the possibility of D1-like receptor involvement in grooming behavior, specific, reversible antagonists of DARs were injected into the CX prior to EEDQ. This procedure was intended to protect D1-like receptors selectively from EEDQ antagonism, thus allowing for selective removal of D2-like receptors by EEDQ. With this rationale, injection of the reversible D2 selective S-sulpiride prior to EEDQ treatment preserved the D2-like receptors, allowing for selective D1-like antagonism through EEDQ treatment. In contrast, injection of the D1-like receptor antagonist SCH23390 prior to EEDQ preserved the D1-like subfamily only. With these procedures, stung cockroaches injected with S-sulpiride prior to EEDQ did not show normal sting-induced grooming behavior. On the contrary, stung cockroaches injected with SCH23390 prior to EEDQ showed normal sting-induced grooming behavior. Together with the previous results, this suggests a major involvement of D1-like receptors with grooming behavior.

The adult *Drosophila* brain exhibits intense D1-like receptor immunoreactivity in the central complex (Kim et al., 2003). More specifically, dDA1 and DAMB (both D1-like) receptors display distinct expression patterns in the CX, with DA1 being the most conspicuous and detected in three out of four CX structures (Kim et al., 2003). Hence, the major role of D1-like receptor in grooming behavior seems to be consistent in other insect species such as fruit flies and bees, as well as in mammals (Wachtel et al., 1992; Mustard et al., 2010; Taylor et al., 2010; Pitmon et al., 2016; Barradale et al., 2017).

According to the results discussed thus far, DAR signaling in stung cockroaches appears to be essential for spontaneous and venom-evoked behavior. After the wasp sting, the escape behavior of cockroaches is also affected. More specifically, during the long term hypokinetic state evoked by the venom, cockroaches, although not paralyzed, fail to escape aversive stimuli (Gal and Libersat, 2008). To investigate whether DARs regulate this behavior as well, cockroaches were subjected to EEDQ injection prior to a wasp sting. The results of this experiment suggest that EEDQ is able to shorten the long-term effects of venom on the escape response. It might be that DARs in cockroaches also are important for mediating the long-term effect of the venom on locomotion. A study of honey bee behavior shows that distinct dopamine signaling pathways mediating D1- and D2-like receptor subtypes in the brain regulate behavioral switching between grooming and walking (Mustard et al., 2010). Likewise, rodents mobilize D1 and D2 receptors signaling for different purposes. It therefore seems possible that D1 signaling is recruited for grooming, while D2 receptors modulate locomotion (Fornaguera et al., 1995; O'Sullivan et al., 2010).

In conclusion, our findings strongly indicate that D1-like receptors are involved in venom-induced grooming behavior exhibited by stung cockroaches. Furthermore, DAR signaling appears to be necessary for induction of long-term hypokinesia by envenomation.

Flies and cockroaches perform distinct bouts of stereotypic grooming movements (Weisel-Eichler et al., 1999; Seeds et al., 2014). Interestingly, rodents utilize the nigro-striatal dopamine system for execution of movement sequences known as syntactic grooming chains. An intact striatum is necessary for correct implementation of grooming chains and electrophysiological recordings from the dorsolateral striatum reveal neurons that encode the entire grooming sequence pattern (Meredith and Kang, 2006). Unilateral 6-hydroxydopamine (6-OHDA) lesion of midbrain DA neurons reduces self-grooming behavior in mice (Pelosi et al., 2015). Moreover neural activity in the pars reticulata region of the substantia nigra appears to promote initiation of the grooming pattern (Meyer-Luehmann et al., 2002). This suggests that basal ganglia play coordinated roles in both initiation and organization of locomotion and grooming. Likewise, in the present investigation and previously published studies, we show that the CX is involved in both initiation of locomotion (Kaiser and Libersat, 2015) and grooming. Moreover, dysfunction in both mammalian basal ganglia and the insect CX results in behavioral defects, including motor abnormalities. Finally, the ontogeny of basal ganglia and the CX share underlying developmental genetic programs from homologous genes to patterned expression and function (Fiore et al., 2015). This suggests a deep homology shared by the insect central complex and the vertebrate basal ganglia circuitries underlying selection and maintenance of behavioral actions (Strausfeld and Hirth, 2013). The present study provides further support for this functional homology and implicates a mechanism for motor control that transcends phylogenetic borders.
